# New York Academy of Medicine

**Published:** 1876-06

**Authors:** Isaac E. Taylor

**Affiliations:** Chairman


New York Academy of Medicine—*Prof. Isaac E. Taylor, Chairman.*—A stated meeting was held at the residence of the Chairman, March 20th, 1876.

Prof. Taylor, with brief and appropriate remarks, welcomed his guests, thanked the members for their suffrages in making him their presiding officer, and pointed out what should be accomplished by the section. The regular order for the evening being *phantom tumors.*

Dr. Chadsey referred to a condition which he had met in a woman forty years of age, who had never borne children. The lower part of the abdomen was distended by a hard tumor, that pressed upon the bladder to such an extent as to render catherization necessary. The bladder being emptied, an active cathartic and emetic were administered; the catamenia appeared, not very profusely, and the uterine tumor was reduced to its natural size. The enlargement had been felt by the woman for some time, but with the return of the menstrual flow had entirely disappeared. Inquiry: Was it congestion of the uterus, or something else?

Dr. Worster remarked that he had seen cases in which venous congestion, depending upon flexions, had been developed to such an extent as to give rise to a condition closely resembling what is commonly understood by the word tumor, but which had entirely disappeared when the neck of the uterus had been straightened and a certain amount of local dissection made. He referred to one case in which the pelvis was filled with a uterus that was enlarged in consequence of arrested return circulation dependent upon flexion. The flexion removed, the parts were restored to their normal size.

Dr. Downs referred to a case which he thought might be classed under the head of *unusual uterine hemorrhage.*

He was called to visit an unmarried German girl, æt. 28 years, and found her pale and almost pulseless, on acconnt of loss of blood from the uterus. A piece of ice, as large as could be used, was at once introduced into the vagina and carried up to the cervix. The os was open to about half the size of the index finger, a little soft about the edges, and situated to the left side of the pelvis. The body of the uterus could be easily recognized lying to the right side of the pelvis, and when pressed upon gave a sensation to the finger as though it contained fluid. A bandage was adjusted to retain the ice in position, and drachm doses of equal parts of fluid extract of ergot and paregoric were administered every half hour until the flow ceased.

The blood which was lost clotted quickly, was of a bright red color, and was something more than three quarts by measure. It was closely examined, but no evidences were found which led the doctor to believe that the flow depended upon a miscarriage.

The woman had not had her courses as freely for three or four months as usual; otherwise they had been regular.

At the end of two weeks the doctor was not able to make anything more of the case, and came to regard it as one of uterine congestion, which relieved itself suddenly in the way of this profuse flow.—*Medical Record*, April.

